# Podoconiosis in Uganda: prevalence, geographical distribution and risk factors

**DOI:** 10.1093/trstmh/trae046

**Published:** 2024-08-14

**Authors:** Ivan Masete, Hope Simpson, Gabriel Matwale, Francis Mutebi, Marlene Thielecke, Fred Nuwaha, George Mukone, Kebede Deribe, Gail Davey

**Affiliations:** Research Department, Innovations for Tropical Disease Elimination, Mukono, P.O. Box 24461, Kampala, Uganda; Department of Global Health and Infection, Brighton and Sussex Medical School, 94 N - S Rd, Falmer, Brighton, BN1 9PX, UK; Vector-borne and Neglected Tropical Diseases Division, Ministry of Health, P.O. Box 1661, Kampala, Uganda; College of Veterinary Medicine, Animal Resources and Biosecurity, Makerere University, P.O. Box 7062, Kampala, Uganda; Charité Center for Global Health, Institute of International Health, Charité University Medicine, 13353 Berlin, Germany; Department of Disease Control and Environmental Health, Makerere University School of Public Health, P.O. Box 7062, Kampala, Uganda; Research Department, Innovations for Tropical Disease Elimination, Mukono, P.O. Box 24461, Kampala, Uganda; Department of Global Health and Infection, Brighton and Sussex Medical School, 94 N - S Rd, Falmer, Brighton, BN1 9PX, UK; Children's Investment Fund Foundation, Addis Ababa, Ethiopia; Department of Global Health and Infection, Brighton and Sussex Medical School, 94 N - S Rd, Falmer, Brighton, BN1 9PX, UK; School of Public Health, College of Health Sciences, Addis Ababa, University, Addis Ababa, Ethiopia

**Keywords:** lymphoedema, neglected tropical diseases, podoconiosis, prevalence, risk factors

## Abstract

**Background:**

Podoconiosis is a neglected debilitating yet preventable disease. Despite its public health significance, podoconiosis is often misdiagnosed and confused with lymphatic filariasis. No appropriate diagnostic tests exist, contributing to underestimation and the absence of control interventions.

**Methods:**

A population-based cross-sectional survey was conducted in seven districts with suspected or reported cases of podoconiosis or an altitude of 1200 m above sea level. Conducted from 30 January to 19 March 2023, the survey employed multilevel stratified sampling to reach eligible household members.

**Results:**

Of the 10 023 participants sampled, 187 (confidence interval 1.25 to 2.78) had clinical features of podoconiosis. The highest prevalence was recorded in Nakapiripirit (7.2% [58/809]) and Sironko (2.8 [44/1564]) and the lowest in Kasese (0.3% [5/1537]), but ranged from 1.1 to 1.8% in Zombo, Rukungiri, Gomba and Hoima districts. The duration of podoconiosis was reported to range from 1 to 57 y. Factors associated with podoconiosis occurrence included advanced age, tungiasis, household cleanliness and personal hygiene. Sleeping on a bed, bathing daily, use of soap and use of footwear in at least moderate condition were protective against podoconiosis.

**Conclusions:**

Podoconiosis occurred in all the sampled districts and was linked to personal hygiene. Long-standing cases suggest an absence of treatment. There is potential for early intervention using a holistic care model in managing this condition. Urgent action and stakeholder engagement are essential for effective podoconiosis management.

## Introduction

Podoconiosis is a non-filarial, non-infectious lymphoedema of the lower limb. The aetiology of the disease is multifactorial, involving a complex interplay between genetic predisposition, environmental exposure and behavioural factors. Barefoot exposure to alkalic red clay soils formed from volcanic base rock under specific environmental circumstances (high altitude and rainfall) over a period of at least 10 y^1^ leads to progressive oedema in genetically susceptible people.^2^ Mineral particles that penetrate the skin cause inflammation and fibrosis of the lymphatic vessels, obliterating the lumen, resulting in blockage of lymphatic drainage. This leads to swelling in the feet and legs, which then advances to lymphoedema and changes in the skin, characterized by nodules.^3^ Clinically, the disease presents with progressive bilateral, but often asymmetric, swelling of the legs.

Limited clinical suspicion and the absence of a positive diagnostic test for podoconiosis have contributed to the underestimation of its prevalence in the past. The diagnosis of podoconiosis and its differentiation from other forms of lymphoedema, relies on a combination of diagnostic tools. These include a thorough medical history assessment, evaluation of chronic diseases, detailed physical examination and, in some cases, laboratory confirmation. In addition, the disease is staged using a clinical staging system that incorporates features related to the disease's duration, severity, complications and regression with treatment.^4^

Podoconiosis is often confused with lymphatic filariasis (LF), another major cause of lymphoedema in the tropics.^4^ LF is caused by thread-like nematodes of the family Filarioidea and is transmitted to humans by mosquitoes. In Uganda, LF is exclusively caused by *Wuchereria bancrofti*, transmitted by *Anopheles* mosquitoes.^5^ LF can be asymptomatic, acute or chronic. Chronic LF leads to lymphoedema, elephantiasis and hydrocele.^6^ Like podoconiosis, LF results in lifelong physical disability and stigma and is strongly associated with poverty. In Uganda, LF has been documented in areas in the north, east and Busoga Region (south of Lake Kyoga) and a small focus in western Uganda.^7–10^ It is estimated that >14 million people are at risk of acquiring the infection. However, by 2020, all at-risk districts in Uganda were under surveillance as a last step towards certification for elimination,^11^ with zero cases reported in two previous national LF surveys.

Podoconiosis mapping has been conducted in some endemic countries,^8,12–14^ and suitability for the disease has been predicted using survey data and environmental variables.^13^ Evidence consensus mapping suggests that Uganda is among the countries with the highest burden of podoconiosis,^15^ although representative prevalence estimates are lacking for much of the country. A previous study identified a prevalence of 450 per 10 000 in three villages of Kapchorwa district^8^ and another study reported prevalence rates of 13 per 10 000 and 6.5 per 10 000 in Busiriba and Kamwenge, respectively, two subcounties of Kamwenge district.^16^ Other areas where cases of podoconiosis have been documented include Kabale in southwestern Uganda^17^ and Napak district in the Karamoja Region.^18^

Although podoconiosis is not included in the World Health Organization (WHO) list of neglected tropical diseases (NTDs), it shares the defining characteristics of this disease group, being strongly associated with poverty and underresourced relative to its burden.^19^ It has been included in the NTD masterplans for endemic countries including Ethiopia^20^ and Rwanda.^21^ The WHO recommends integration of case finding, control and management of NTDs where their distributions overlap.^22^ Surveys and case management interventions have been successfully integrated for podoconiosis and LF in Ethiopia,^23,24^ and tungiasis may be a suitable target for integrated control with podoconiosis given their shared risk factors.

Currently, Uganda lacks government guidelines for the treatment and control of podoconiosis, with minimal interventions against the disease. This is attributed, in part, to a limited understanding of the disease's geographical distribution. Despite this, podoconiosis is recognized as one of the NTDs of the highest public health significance in Uganda, as outlined in the Sustainability Plan for the Neglected Tropical Diseases Control Program (2020–2025).

In this study we aim to contribute to a better understanding of the spatial distribution, burden and risk factors of podoconiosis in Uganda.

## Methods

### Study area

The study was conducted in seven districts of Gomba, Hoima, Kasese, Nakapiripirit, Rukungiri, Sironko and Zombo. The districts exhibit diverse characteristics across climate, livelihoods and cultural practices. In Rukungiri, Kasese and Hoima in the Western Region, the climate is generally characterized by moderate temperatures and ample rainfall, supporting agriculture as a primary livelihood. People in these areas predominantly engage in subsistence farming and animal husbandry, with a focus on crops such as coffee, tea and livestock rearing.

Sironko, in the Elgon Region, experiences a varied climate due to its topography. People here rely on mixed agriculture, cultivating crops like maize, beans and coffee. Animal husbandry is also common, contributing to the local economy. In Zombo, located in the West Nile Region. People rely on agriculture, with crops like cassava and millet being staples. Gomba, in the Central Region, experiences a favourable climate for agriculture. People engage in subsistence farming, growing crops like bananas, maize and beans. Animal husbandry, especially cattle rearing, is an integral part of the local economy. Nakapiripirit, in the Karamoja Region, has two ecological zones. The northern part of the district has a semi-arid climate, influencing a pastoralist lifestyle. The community depends heavily on livestock, primarily cattle, and practices transhumance to find suitable grazing areas. The southern part of the district has a connection with the Mount Elgon ranges, where agriculture is intensely practiced.

While each region has its unique characteristics, similarities lie in the reliance on agriculture and animal husbandry for livelihoods. Differences emerge in the specific crops grown, the nature of animal husbandry and the adaptation strategies to the varying climates, reflecting the rich cultural and environmental diversity across these districts.

### Study design

A population-based cross-sectional survey was conducted between 30 January and 19 March 2023 in selected districts that met the following inclusion criteria: having suspected or reported cases of podoconiosis, being situated at an altitude of ≥1200 m above sea level, or both, and not having been previously mapped for podoconiosis. Of a total of 45 districts meeting these criteria, 7 were randomly selected: Rukungiri, Kasese and Hoima in Western Region; Sironko in the Elgon Region; Zombo in the West Nile Region; Gomba in the Central Region and Nakapiripirit in the Karamoja Region. The target study population was individuals ≥15 y of age who had lived within the selected districts for at least 10 y.

### Sample size and sampling procedure

The sample size for each district, based on an assumed prevalence of 6%, absolute precision of 2%, expected participation rate of 90% and a design effect of 2.5, was calculated to be 1505 people. Given that the survey covered seven districts, the overall sample size for the study was 10 535. Multilevel stratified sampling was used to select the participants. In each district, six subcounties were randomly selected; from each subcounty, a parish was randomly selected; and from each parish, a village was randomly selected. The probability of inclusion for subcounties, parishes and villages was not proportionate to their size. A minimum of six villages were surveyed in all districts except Nakapiripirit, in which three were surveyed, due to its small and sparse population. At the village level, all households in the area were invited to participate. If the selected village had few respondents, households in the adjacent villages were included until the sample size was achieved. At the household level, all members meeting the inclusion criteria and available at the time of the study were invited to participate.

### Data collection

Data were collected using KoBoCollect on Android tablets, covering demographics, socio-economic information, shoe use and hygiene behaviour. Material composition of dwelling walls, roofs and floors was recorded and cleanliness was assessed through observation for visible dirt or debris in living spaces, as well as assessing the general tidiness and organization of the living area. Disability was determined by inquiring about difficulty walking due to the disease. The condition of footwear was assessed based on the integrity of the sole and the overall structural soundness, looking for damage to the soles and any visible defects.

All participants were examined for tungiasis, scabies, warts, headlice, cutaneous larva migrans and myiasis as possible comorbidities of podoconiosis. Tungiasis was diagnosed by physical examination of the feet and hands. Before the examination, the feet or digits were washed with soap and water. Diagnosis of tungiasis was based on the morphology of embedded sand fleas, as described by Eisele et al.^25^ Embedded sand fleas, which presented as dark brown to black spots in the centre of a hyperaemic rim or yellow to white nodular lesions 2–12 mm in diameter, were categorised as viable. Raised circular brown to black patches or shallow circular skin craters with necrotic edges were characterised as avital lesions. Skin sores from which embedded sand fleas had been completely or partially removed were categorised as manipulated lesions. An individual was classified as affected by tungiasis if he/she had at least one embedded sand flea, whether vital, dead or manipulated.

The study used a screening question for identifying those with visible swelling of the lower leg (lymphoedema). A simple checklist was then used to examine for possible differential diagnoses of podoconiosis. The checklist included consideration of the clinical presentation of podoconiosis and exclusion of other causes of lymphoedema or swelling of the lower legs. The case definition for podoconiosis was swelling of the lower limb with a duration of at least 1 y but not since birth, in a person ≥15 y of age, with no reported groin swelling, no history or signs of heart disease, no previous diagnosis or signs of leprosy (loss of sensation in the toes, anaesthetic skin patches or hypopigmentation) and no other possible causes of lymphoedema (LF, pregnancy, cellulitis, insect bite, allergic reaction, injury). At the time of the study, two national LF surveys had been conducted and they both reported zero cases of the diseases in the study districts. LF was thus differentiated from podoconiosis by comparing disease presentations. Podoconiosis was diagnosed as a lymphoedema that typically starts in the feet, progresses upward and is bilateral in nature, while LF starts from the groin and moves downward. Further, the survey team was thoroughly trained in differentiating the two diseases.

Individuals displaying signs of lower limb swelling were interviewed regarding the duration of their illness and their perceptions of the causes behind their condition. A comprehensive physical examination, which included assessing sensation preservation in the toes, clinical manifestations of podoconiosis and examination for groin involvement, was conducted. The staging of podoconiosis in these cases was determined using a chart based on the clinical staging system proposed by Tekola et al. in 2008.^4^

### Statistical analysis

Data were analysed using Stata software, version 17.0 (StataCorp, College Station, TX, USA). For descriptive statistics, categorical variables were summarised as counts and percentages, while continuous variables were summarised using means and medians. The prevalence of podoconiosis was calculated as the percentage of respondents who were assessed and suspected to have the disease. A χ^2^ test was used to assess the relationship between having podoconiosis and other study variables. Factors associated with podoconiosis were determined using a backward stepwise logistic regression model. For inclusion of variables in the model, a p-value <0.2 was used. However, for the final model, a p-value <0.05 was used to test significance at a 95% confidence interval (CI).

## Results

A total of 10 023 individuals (60.1% [n=6027] female, 39.9% [n=3996] male) were reached and screened in 39 subcounties in seven districts, giving a 95% response rate. The age of the respondents ranged from 15 to 85 y. Almost half (44.9% [n=4497]) had achieved some primary education (nursery–primary 6), but only 14% had completed primary school. Most of the respondents were Christians (91.0% [n=9117]) (Table 1). The majority of the respondents had no skin condition (96.0% [n=9622]) or disability (95.2% [n=9538]). In case of a disability, most had a physical one (90.7% [n=440]).

**Table 1.** Bivariate analysis of the factors associated with occurrence of podoconiosis

	Podoconiosis		Bivariate analysis	
Variable	Yes, n (%)	No, n (%)	OR (95% CI)	p-Value
Education level				
None	78 (43.1)	1421 (14.4)	Ref	
Some primary (Nursery–P6)	85 (47.0)	4412 (44.8)	0.4 (0.3 to 0.5)	<0.001*
Complete primary (sat for PLE)	8 (4.4)	1399 (14.2)	0.1 (0.1 to 0.2)	<0.001*
Some secondary (S1–S5)	10 (5.5)	2136 (21.7)	0.1 (0.04 to 0.2)	<0.001*
Complete secondary (sat for A-level exams)	0	126 (1.3)	1	–
Further	0	348 (3.5)	1	–
Occupation				
Unemployed	41 (22.7)	756 (7.7)	Ref	
Casual labourer	9 (5.0)	293 (3.0)	0.6 (0.3 to 1.2)	0.129*
Crop farmer	100 (55.3)	5848 (59.4)	0.3 (0.2 to 0.5)	<0.001*
Engineer	0	55 (0.6)	1	
Fisherman	4 (2.2)	75 (0.8)	1.0 (0.3 to 2.8)	0.975
Government employee	1 (0.6)	67 (0.7)	0.3 (0.03 to 2.0)	0.206
Healthcare services	1 (0.6)	68 (0.7)	0.3 (0.03 to 2.0)	0.201
Housewife	1 (0.6)	197 (2.0)	0.1 (0.01 to 0.7)	0.020*
Hunter	0	6 (0.1)	1	–
Full-time student	2 (1.1)	789 (8.0)	0.04 (0.01 to 0.2)	<0.001*
Trader	21 (11.6)	1321 (13.4)	0.3 (0.2 to 0.5)	<0.001*
Mechanic	0	56 (0.6)	1	–
Teacher/education	0	232 (2.4)	1	–
Transport	0	54 (0.6)	1	–
Wood cutter	1 (0.6)	25 (0.3)	0.7 (0.1 to 5.6)	0.768
Presence of skin disease				
No	123 (68.0)	9499 (96.5)	Ref	
Yes	58 (32.0)	343 (3.5)	13.1 (9.3 to 18.2)	<0.001*
Water source				
Community borehole/tube well	71 (39.2)	3474 (35.3)	Ref	
Delivered (tank, cart, kiosk)	3 (1.7)	86 (0.9)	1.7 (0.5 to 5.5)	0.372
Dug well (protected)	15 (8.3)	1117 (11.4)	0.7 (0.4 to 1.2)	0.142*
Dug well (unprotected)	19 (10.5)	526 (5.3)	1.8 (1.1 to 3.0)	0.030*
Packaged water	1 (0.6)	47 (0.5)	1.0 (0.1 to 7.7)	0.968
Piped into the dwelling	3 (1.7)	231 (2.4)	0.6 (0.2 to 2.0)	0.445
Piped outside the dwelling	5 (2.8)	345 (3.5)	0.7 (0.3 to 1.8)	0.461
Piped to a neighbour	1 (0.6)	135 (1.4)	0.4 (0.05 to 2.6)	0.315
Public tap or standpipe	23 (12.7)	1740 (17.7)	0.6 (0.4 to 1.0)	0.071*
Surface water (river, dam, lake, pond)	40 (22.1)	2141 (21.8)	0.9 (0.6 to 1.4)	0.653
History of water scarcity				
No	107 (59.1)	7047 (71.6)	Ref	
Yes	74 (40.9)	2795 (28.4)	1.7 (1.3 to 2.4)	<0.001*
Body hygiene				
Fair	102 (56.4)	5627 (57.2)	Ref	
Clean	25 (13.8)	3674 (37.3)	0.4 (0.2 to 0.6)	<0.001*
Dirty	54 (29.8)	541 (5.5)	5.5 (3.9 to 7.7)	<0.001*
Possesses shoes				
No	41 (22.7)	785 (8.0)	Ref	
Yes	140 (77.3)	9057 (92.0)	0.3 (0.2 to 0.4)	<0.001*
Wore shoes during interview				
No	37 (26.4)	2975 (32.9)	Ref	
Yes	103 (73.6)	6082 (67.2)	1.4 (0.9 to 2.0)	0.110
Type of foot wear				
Closed leather	4 (2.9)	447 (5.0)	0.2 (0.04 to 0.8)	0.024*
Closed plastic	3 (2.1)	710 (7.8)	0.1 (0.02 to 0.4)	0.002*
Closed textile (sneakers)	4 (2.9)	215 (2.4)	0.4 (0.1 to 1.7)	0.189*
Sandals	119 (85)	7086 (78.2)	0.3 (0.1 to 1.1)	0.060*
Rubber boots	7 (5.0)	541 (6.0)	0.3 (0.1 to 1.0)	0.049*
Other	3 (2.1)	58 (0.6)	Ref	
Frequency of wearing shoes				
3 to 5 times/week	11 (7.9)	637 (7.0)	Ref	
Every day	99 (70.7)	6994 (77.2)	0.8 (0.4 to 1.5)	0.535
<3 times/week	12 (8.6)	207 (2.3)	3.4 (1.5 to 7.7)	0.004*
Only for special occasions	12 (8.6)	1179 (13.0)	0.6 (0.3 to 1.3)	0.208
Sometimes per month	6 (4.3)	40 (0.5)	8.7 (3.1 to 24.7)	<0.001*

*p<0.2.

Ref: reference.

### Prevalence of podoconiosis

Of the 10 023 participants, 189 individuals (1.9%) with physical signs of lymphoedema on the lower limbs were identified and clinically assessed and 181/189 (95.8%) were found to have podoconiosis. The overall estimated prevalence of podoconiosis was 1.81% (95% CI 1.55 to 2.09) (Table 2). Males had a marginally higher prevalence of podoconiosis (2.9%) compared with females (1.6%). The mean age of podoconiosis cases was 52.4±20.7 y. The prevalence of podoconiosis increased with age (Table 3), starting from 0.6% in the 15–24 y age group and reaching 5.8% in individuals ≥65 y, with a notable increase in prevalence after the age of 45 y, indicating that older age groups were more susceptible to podoconiosis. Occupation analysis revealed that the majority of the 187 podoconiosis cases were crop farmers (55.6% [n=104]), followed by unemployed (21.9% [n=41]), traders (12.3% [n=23]) and casual labourers (4.8% [n=9]). The remaining 5.4% (n=10) included fishermen, office workers, a nurse, students, a housewife and a wood cutter.

**Table 2.** Prevalence of podoconiosis by district

District	Patients examined, n	Podoconiosis cases, n	Prevalence, % (95% CI)
Nakapiripirit	809	57	7.05 (5.38 to 9.03)
Sironko	1564	41	2.62 (1.89 to 3.54)
Hoima	1535	26	1.69 (1.11 to 2.47)
Rukungiri	1531	18	1.18 (0.70 to 1.85)
Gomba	1519	17	1.12 (0.65 to 1.79)
Zombo	1532	17	1.11 (0.65 to 1.77)
Kasese	1537	5	0.33 (0.11 to 0.76)
Total	10 027	181	1.81 (1.55 to 2.09)

**Table 3.** Factors associated with the occurrence of podoconiosis

	Podoconiosis		Bivariate analysis		Multivariate analysis	
Variable	Yes, n (%)	No, n (%)	OR (95% CI)	p-Value	AOR (95% CI)	p-Value
Age group (years)						
15–24	21 (0.6)	3464 (99.4)	Ref	–	Ref	–
25–34	24 (1.3)	1890 (98.7)	2.1 (1.2 to 3.8)	0.014*	2.1 (1.0 to 4.2)	0.037*
35–44	20 (1.3)	1494 (98.7)	2.2 (1.2 to 4.1)	0.012*	2.4 (1.2 to 4.9)	0.015*
45–54	29 (2.2)	1283 (97.8)	3.7 (2.1 to 6.6)	<0.001*	3.7 (1.9 to 7.3)	<0.001*
55–64	29 (6.7)	765 (96.3)	6.2 (3.5 to 11.0)	<0.001*	7.3 (3.7 to 14.3)	<0.001*
≥65	58 (5.8)	945 (94.2)	10.1 (6.1 to 16.8)	<0.001*	8.1 (4.3 to 15.1)	<0.001*
Tungiasis						
No	151 (1.5)	9792 (98.5)	Ref	–	Ref	–
Yes	30 (35.7)	54 (64.3)	36.0 (22.4 to 57.9)	<0.001*	7.5 (3.5 to 16.1)	<0.001*
Room hygiene						
Clean	20 (0.6)	3391 (99.4)	Ref	–	Ref	–
Fair	111 (1.9)	5847 (98.1)	3.2 (2.0 to 5.2)	<0.001*	2.2 (1.3 to 3.9)	0.004*
Very dirty	50 (7.7)	604 (92.4)	14.0 (8.3 to 23.7)	<0.001*	3.0 (1.5 to 6.3)	0.002*
Sleep on a bed						
No	104 (3.9)	2564 (96.1)	Ref	–	Ref	–
Yes	77 (1.0)	7278 (99.0)	0.3 (0.2 to 0.4)	<0.001*	0.4 (0.3 to 0.6)	<0.001*
Bathing frequency						
Less often	14 (12.8)	95 (87.2)	Ref	–	Ref	–
Once a day	72 (2.3)	3113 (97.7)	0.2 (0.1 to 0.3)	<0.001*	0.3 (0.1 to 0.8)	0.013*
Once a week	8 (13.6)	51 (86.4)	1.1 (0.4 to 2.7)	0.896	0.3 (0.1 to 1.4)	0.112
Several times per week	14 (9.2)	138 (90.8)	0.7 (0.3 to 1.5)	0.352	0.9 (0.3 to 2.7)	0.846
Twice or more per day	73 (1.1)	6445 (98.9)	0.1 (0.04 to 0.1)	<0.001*	0.2 (0.1 to 0.6)	0.003*
Use of soap						
Always	93 (1.3)	6980 (98.7)	Ref	–	Ref	–
No	25 (15.7)	134 (84.3)	14.0 (8.7 to 22.5)	<0.001*	2.8 (1.3 to 6.3)	0.010*
Sometimes	63 (2.3)	2728 (97.7)	1.7 (1.3 to 2.4)	0.001*	0.8 (0.6 to 1.3)	0.439
Footwear condition						
Broken	43 (9.2)	427 (90.8)	Ref	–	Ref	–
Moderate	69 (1.2)	5798 (98.8)	0.1 (0.1 to 0.2)	<0.001*	0.2 (0.2 to 0.4)	<0.001*
Very good	28 (1.0)	2832 (99.0)	0.1 (0.1 to 0.2)	<0.001*	0.3 (0.2 to 0.6)	<0.001*

*p<0.05.

Ref: reference.

### Clinical characteristics of podoconiosis

The duration of podoconiosis among cases ranged from 1 to 57 y. Most of the cases (118 [65.2%]) were in the first decade of the disease, followed by 38 (21.0%) in the second and 25 (13.8%) the third and beyond. The majority had bilateral swelling (70.7% [n=128]), most of which was ascending (86.7% [n=157]) and asymmetrical (69.6% [n=126]).

The most common symptoms (from which respondents could select multiple options) were pain in the legs, skin itching and burning sensations. For the right leg, the mean midfoot circumference was 25.9±5.8 cm and the mean midcalf circumference was 30.8±21.9 cm.

The median number of acute lymphatic attack episodes reported in the last 3 months was 5 (interquartile range 2–10). A total of 54.1% (98 cases) exhibited a foul odour, indicative of fungal or bacterial infection of one or both legs. Interdigital maceration was observed on both legs in 45.9% (n=83) of cases. A significant proportion of individuals reported limitations in their daily activities: 39.8% (n=72) were greatly limited, 27.1% (n=49) were limited, 26.0% (n=47) were slightly limited and only 7.1% (n=13) experienced no limitations at all. Hyperkeratosis was found in both legs in 61.3% (n=111) of cases. The presence of pain showed a significant association with the restriction of daily activities (p<0.001).

The disease was staged based on the proximal spread of swelling and dermal nodules, bands and ridges.^4^ The proximal spread was measured in relation to easily identifiable landmarks (below or above the knee and below or above the ankle). Among the 181 cases, 79.6% exhibited swelling below the knee, 2.8% above the knee, 2.8% both above and below the knee and 14.9% across the entire foot. Consequently, the majority of cases were classified as stage 1 (46.4% [n=84]), followed by stage 2 (28.7% [n=52]) and stage 3 (17.1% [n=31]), with stages 5 (5.5% [n=10]) and 4 (2.2% [n=4]) representing a smaller proportion. Most individuals in stages 1–3 had the disease for 0–10 y, while those in stage 5 typically had it for >11 y. A significant association between disease stage and the duration of illness was observed (p=0.001).

### Spatial distribution of podoconiosis

Podoconiosis prevalence was highest in the eastern districts of Nakapiripirit (7.2%) and Sironko (2.8%) (Table 2). Podoconiosis prevalence in Zombo, Rukungiri, Gomba and Hoima ranged from 1.1 to 1.8% and was lowest in Kasese (0.3%).

While the study was not powered to estimate community-level prevalence with accuracy, there was no obvious clustering of podoconiosis cases at the community level. Community-level prevalence of podoconiosis ranged from 0 to 7.1%, with the highest prevalence identified in a community of Nakapiripirit, where 58 cases were identified out of a sample of 809 people.

### Factors associated with podoconiosis

After adjusting for variables significantly associated with podoconiosis in the bivariate analysis (Table 1), such as education level, the presence of a skin condition, water source, history of water scarcity, body hygiene, possession of shoes, wearing of shoes during the interview, type of footwear, age of first footwear use and frequency of wearing shoes, the following factors were significantly linked to podoconiosis: age group, disability status, comorbidity with tungiasis, room hygiene, frequency of body and feet washing, soap use during bathing and the condition of footwear.

The risk of podoconiosis increases with age, with those ≥65 y of age being the most vulnerable (adjusted odds ratio [AOR] 8.1 [95% CI 4.3 to 15.1], p<0.001). Podoconiosis was also more likely among individuals with tungiasis (AOR 7.5 [95% CI 3.5 to 16.1], p<0.001). Moreover, it was more prevalent among individuals residing in very unclean rooms (AOR 3.0 [95% CI 1.5 to 6.3], p=0.002) and those who do not use soap during bathing (AOR 2.8 [95% CI 1.3 to 6.3], p=0.010). The results are presented in Table 3.

Conversely, protective factors against podoconiosis include sleeping on a bed (AOR 0.4 [95% CI 0.3 to 0.6], p<0.001), bathing once a day (AOR 0.3 [95% CI 0.1 to 0.8], p=0.013) or twice or more a day (AOR 0.2 [95% CI 0.1 to 0.6], p=0.003) and footwear in moderate (AOR 0.2 [95% CI 0.2 to 0.4], p<0.001) or very good condition (AOR 0.3 [95% CI 0.2 to 0.6], p<0.001). The results are presented in Table 3.

### Other skin conditions

The study found that 401 (4.0%) of the respondents had a skin condition. Of these, 84 (21.0%) had tungiasis, 55 (13.7%) scabies, 19 (4.7%) warts, 15 (3.7%) headlice, 15 (3.7%) cutaneous larva migrans and 3 (0.8%) myiasis. A total of 207/401 (51.6%) respondents had only one skin condition, 169 (42.1%) had two, 23 (5.7%) had three and 3 (0.7%) had four.

## Discussion

Evidence consensus mapping identifies Uganda as one of the countries with the highest prevalence of podoconiosis.^26^ However, there is a scarcity of data regarding the distribution of podoconiosis in Uganda, hindering the national response to the disease. This study was the first population-based prevalence survey to be conducted across multiple districts in Uganda, offering insights into the spatial distribution and prevalence of podoconiosis. Our findings reveal the overall estimated prevalence of podoconiosis was 1.81 (95% CI 1.55–2.09). Podoconiosis was found to be widespread, but with regional disparities, and affecting a wide demographic range. This information can inform targeted public health interventions and resource allocation in addition to further research and intervention in Uganda.

Previous efforts to map the disease in Uganda were limited to a few districts or regions. From these efforts, podoconiosis was documented in Kapchorwa and Kween,^8^ Kamwenge,^16^ Kabale in western Uganda and Napak in northeastern Uganda.^18^ In these areas, prevalence ranging from 0.13 to 4.5% was reported. The prevalence reported by this study is within this range. However, the earlier studies targeted communities with known cases while this study randomly sampled respondents from at-risk districts, so as to capture a heterogenous sample from across the country. The prevalence rates observed in this study are also comparable to those reported in Cameroon,^27^ which ranged from 0.2 to 2.7%, with a nationwide distribution.

Half of the identified podoconiosis cases reported having the disease for at least 6 y and the most common symptoms included bilateral swelling, pain, itching and burning sensations in the legs. The majority of cases were in the early stages of the disease, meaning they have a good chance to arrest progression to the advanced stages with appropriate and timely intervention. These interventions include reducing contact with soil (through the use of footwear and covering earth floors), foot hygiene and care and exercises to reduce the oedema. Despite most cases being in the early stages of the disease, the majority reported a negative impact of the condition on their quality of life and daily activities, highlighting the urgent need for a multisectoral response. From experience in other countries in which podoconiosis is a significant public health issue, this would include community-based education,^28^ early detection, symptom relief, holistic care,^29^ resource allocation, income-generating activities and policy development. The holistic care model must be integrated into the Ugandan healthcare system to effectively address the complex challenges posed by podoconiosis.

The presence of comorbidities alongside podoconiosis may complicate diagnosis and management, necessitating a holistic approach to healthcare. The relationship between podoconiosis and tungiasis is yet to be fully understood. While it is possible that tungiasis has a role in facilitating entry of soil particles through the wounds and inflammation left behind by extracted and dead sand fleas, the soil particles linked to podoconiosis are small enough to pass through intact skin. It is therefore possible that the lymphoedematous tissues found in people with podoconiosis facilitate sand flea entry. Once established, it is possible that the *Wolbachia* spp. harboured by sandfleas^30^ exacerbate inflammation and thus swelling of the limbs.

## Study Strengths and Limitations

The study derives its strengths from a large sample size of 10 035 respondents and high response rate (95%). However, limitations include that while the sample size was relatively large, it was drawn from only 7 of the 45 at-risk districts, limiting the generalizability of the findings to the entire at-risk population; women were more readily available for interviews than men at the household level; reliance on clinical examination rather than laboratory testing to exclude LF; and possible symptom reporting bias. Upon identification of physical signs of swelling (lymphoedema) of the lower limbs, the suspected cases were taken through a series of questions to inform the clinical examination as well as physical examinations to confirm some of the questions asked.

## Conclusions

In conclusion, this study provides evidence of widespread podoconiosis distribution, a prevalence of >1% and the considerable duration of disease among affected individuals in Uganda. The findings emphasize the potential for early intervention and the significance of a holistic care model in managing this condition. Addressing not only the disease's physical symptoms, but also its impact on the quality of life calls for a multifaceted approach. Moreover, the identification of associated comorbidities, particularly tungiasis, emphasizes the need for an integrated healthcare strategy to effectively manage and mitigate the challenges posed by podoconiosis in Uganda. Based on the findings, it is crucial to mobilize action and engage stakeholders to address the burden of this neglected, yet easily preventable and treatable disease.

Also, due to the limitations posed by the relatively small geographical area sampled, caution should be exercised when extrapolating these results to a broader context. Future research endeavours should aim to include a more extensive sample, encompassing a broader geographical scope, to enhance the robustness and applicability of the findings to the larger population at risk.

## Data Availability

The data set analyzed for this study is available upon reasonable request from the corresponding author.
