# Erratum to: Podoconiosis in Uganda: prevalence, geographical distribution and risk factors

**DOI:** 10.1093/trstmh/trae100

**Published:** 2024-10-30

This is an erratum to: Ivan Masete, Hope Simpson, Gabriel Matwale, Francis Mutebi, Marlene Thielecke, Fred Nuwaha, George Mukone, Kebede Deribe, Gail Davey, Podoconiosis in Uganda: prevalence, geographical distribution and risk factors, *Transactions of The Royal Society of Tropical Medicine and Hygiene*, 2024; trae046, https://doi.org/10.1093/trstmh/trae046

Since the originally published version of this paper, an error in the order of authors has been corrected. Ivan Masete is the first author, Hope Simpson is the second author, and Gail Davey is the last author.

In addition to this, errors in the linking of author affiliations in the paper have also been corrected.

